# Growth of Human Beings

**Published:** 1884-05

**Authors:** 


### THE GROWTH OF HUMAN BEINGS.

THE investigations of the Anthropometric Committee of the British Association, have made more or less clear, several interesting facts respecting the rate of growth of the two sexes. The period of most rapid growth is from birth to five years of age, and then both sexes grow alike, the girls being a little shorter and lighter than the boys. From five to ten the boys grow a little faster than the girls, but from ten to fifteen the girls grow the faster, and at between eleven and a-half and fourteen and a-half years old, are actually taller, and from twelve and a-half to fifteen and a-half are heavier than the boys. The boys, however, take the lead between fifteen and twenty years, and grow at first rapidly, but afterwards slower, and complete their growth at about twenty-three years, while girls grow very slowly after fifteen years of age, and attain their full stature at about the twentieth year. The tracings and tables show a slow but steady increase of stature up to the fiftieth year, and a more rapid increase in weight up to the sixtieth year in men, but the statistics of women are too few after the age of twenty-three to determine the stature and weight of their sex at the more advanced periods of life. The fact that man continues to grow in stature up to his fiftieth year contradicts the popular notions on the subject—according to which he ceases to grow before reaching half that age.

